# Association of *miR-146a, miR-149* and *miR-196a2* polymorphisms with neuroblastoma risk in Eastern Chinese population: a three-center case–control study

**DOI:** 10.1042/BSR20181907

**Published:** 2019-06-07

**Authors:** Chunlei Zhou, Yingzi Tang, Jinhong Zhu, Lili He, Jinghang Li, Yizhen Wang, Haixia Zhou, Jing He, Haiyan Wu

**Affiliations:** 1 Department of Pathology, Children’s Hospital of Nanjing Medical University, Nanjing 210008, Jiangsu, China; 2 Department of Clinical Laboratory, Molecular Epidemiology Laboratory, Harbin Medical University Cancer Hospital, Harbin 150040, Heilongjiang, China; 3 Department of Cardiovascular Surgery, The First Affiliated Hospital of Nanjing Medical University, Nanjing 210029, Jiangsu, China; 4 Department of Pathology, Anhui Provincial Children’s Hospital, Hefei 230051, Anhui, China; 5 Department of Hematology, The Second Affiliated Hospital and Yuying Children’s Hospital of Wenzhou Medical University, Wenzhou 325027, Zhejiang, China; 6 Department of Pediatric Surgery, Guangzhou Institute of Pediatrics, Guangzhou Women and Children’s Medical Center, Guangzhou Medical University, Guangzhou 510623, Guangdong, China

**Keywords:** Neuroblastoma, pre-miRNAs, polymorphisms, susceptibility

## Abstract

Neuroblastoma is one of the most common malignancy in childhood, which originates from the developing sympathetic nervous system. Single nucleotide polymorphisms (SNPs) in primary miRNA (pri-miRNA) have shown to associate with cancer susceptibility, including neuroblastoma. Three precursor miRNA (pre-miRNA) SNPs (*pre-miR-146a* rs2910164, *pre-miR-149* rs2292832 and *pre-miR-196a2* rs11614913) were found to contribute to pathogenesis of various diseases. Here, to evaluate the association among these three pre-miRNA SNPs and neuroblastoma susceptibility in Eastern Chinese children, we carried out a three-center case–control study involving 312 neuroblastoma cases and 762 healthy controls. Odds ratios (ORs) and 95% confidence intervals (CIs) were used to assess the association of these three polymorphisms with neuroblastoma risk. However, no significant association was observed among these three SNPs and neuroblastoma susceptibility, in either overall or subgroups analysis by tumor sites, gender and age. Further larger studies consisting of diverse ethnic populations are required to clarify the associations among these three pre-miRNAs polymorphisms and neuroblastoma risk.

## Introduction

Neuroblastoma, a common childhood extracranial tumor, originates from the developing sympathetic nervous system [1]. It mainly occurs in infancy, with the median age of 17 months at diagnosis [2]. In addition, neuroblastoma accounts for approximately 10% of all malignancies and 15% of malignancy deaths in children [3]. This type of childhood malignancy shows great heterogeneity in clinical presentation and prognosis. Neuroblastoma could be classified into low, intermediate, and high risk groups based on clinical and biological characteristics, including tumor stage, histopathology, age, DNA index, and MYCN amplification [4–6].

Moreover, several genome-wide association studies (GWASs) have identified extra neuroblastoma susceptibility genes, including *CASC15, BARD1, LMO1, DUSP12, HACE1, LIN28B, CPZ* and *RSRC1* [7–12]. Beside GWASs, candidate gene approaches also identified several candidate loci, such as, the *NEFL* rs1059111 polymorphism correlates with increased levels of *NEFL*, and confers protection against neuroblastoma development [13]. The *CDKN1B* rs34330 polymorphism acts as a genetic risk factor for neuroblastoma by affecting the transcription rate [14]. The *BARD1* rs17489363 and rs1048108 polymorphisms could influence neuroblastoma susceptibility [15].

MicroRNAs (miRNAs) are short non-coding RNAs (approximately 17–22 nucleotides in length. These small RNAs can bind to the 3′-untranslated region (3′-UTR) of gene transcripts to induce target mRNA degradation or inhibit translation [16]. miRNAs play important roles in carcinogenesis, including neuroblastoma [17–20]. DNA sequences encoding miRNAs include single miRNA gene loci, miRNA gene clusters, or fragments within introns of protein-coding genes. The primary miRNA (pri-miRNA) are processed to generate small mature miRNAs by the RNase III enzymes, Dicer and Drosha [21]. The single nucleotide polymorphisms (SNPs) in precursor miRNA (pre-miRNA) encoding sequences may influence miRNA expression and maturation, thereby modifying susceptibility to neuroblastoma and other cancers [22–26].

*Pre-miR-146a, pre-miR-149* and *pre-miR-196a2* have been reported as key factors for carcinogenesis due to their targeting on several vital genes [27,28]. The *pre-miR-146a* rs2910164, *pre-miR-149* rs2292832 and *pre-miR-196a2* rs11614913 polymorphisms could influence their functions through altering miRNA maturation, expression, and/or efficiency of targeting [29]. Previous studies showed that *miR-146a* was related to regulation of TNF-α [30], *miR-149* could regulate the expression of MTHFR [31], and *miR-196a2* could target *ANXA1, DFFA* and *PDCD4* [32]. These three well-known pre-miRNAs polymorphisms have been reported to be associated with the pathogenesis of various diseases, such as breast cancer, colorectal cancer, hepatocellular carcinoma, and gastric cancer [33–38]. However, in our previous studies in Chinese children of Southern and North China, no significant association among these three pre-miRNAs’ polymorphisms and neuroblastoma susceptibility was observed [25]. Herein, we performed a three-center hospital-based case–control study to test the association among these three pre-miRNAs’ SNPs and neuroblastoma risk with 312 cases and 762 controls residing in East China.

## Materials and methods

### Study subjects

Participants were recruited from three hospitals: Children’s Hospital of Nanjing Medical University (158 cases and 426 controls, Jiangsu province, China) [39], Anhui Provincial Children’s Hospital (119 cases and 264 controls, Anhui province, China) [40], and The Second Affiliated Hospital and Yuying Children’s Hospital of Wenzhou Medical University (36 cases and 72 controls, Wenzhou area) [41,42]. A total of 313 cases and 762 controls were enrolled for the case–control study (Supplementary Table S1). The study was approved by the institutional review boards of Children’s Hospital of Nanjing Medical University, Anhui Provincial Children’s Hospital and The Second Affiliated Hospital and Yuying Children’s Hospital of Wenzhou Medical University. The selection standard and details of the included participants are accessible in our previous studies [43–46]. All participants or their guardians provided written informed consent before the study.

### SNPs selection and genotyping

Three well-known polymorphisms (*miR-146a* rs2910164, *miR-149* rs2292832 and *miR-196a2* rs11614913) were selected. An online tool, SNPinfo (http://snpinfo.niehs.nih.gov/) was used to predict the functions of selected SNPs (Supplementary Table S2). DNA samples were extracted as previously described [45]. Briefly, DNA samples were diluted to a stock concentration of 10 ng/μl and added to 96-well plates. Genotyping for these three polymorphisms was carried out in a 384-well plate using TaqMan Real-Time PCR method [47,48]. Moreover, 10% of the samples were randomly chosen for duplicate analyses and the results of quality control samples were 100% concordant.

### Statistical analysis

Deviation from Hardy–Weinberg equilibrium (HWE) in controls was calculated for the selected SNPs by goodness-of-ft χ^2^ test. Differences in the genotypes and demographics between cases and controls were assessed by χ^2^ test. To evaluate the association among the three selected SNPs and neuroblastoma susceptibility, odds ratios (ORs) and 95% confidence intervals (CIs) were calculated using a multivariate logistic regression. Moreover, stratified analyses were evaluated by tumor sites, gender and age. A *P*<0.05 was considered as statistically significant. All statistical tests were calculated using SAS software (v9.4; SAS Institute, Cary, NC, U.S.A.).

## Results

### Participants’ characteristics

The clinical and demographic characteristics of 313 neuroblastoma cases and 762 controls are shown in Supplementary Table S1. There were no significant differences between neuroblastoma cases and controls in age (*P*=0.823) and gender (*P*=0.610).

### Association between selected *miRNA* polymorphisms and neuroblastoma risk

As shown in Table 1 and Supplementary Table S3, the genotype counts and association results of these three pre-miRNAs’ polymorphisms (*pre*-*miR-146a* rs2910164, *pre*-*miR-149* rs2292832 and *pre*-*miR-196a2* rs11614913) were assessed. All of the three selected SNPs were in accordance with the HWE in controls. However, no significant association was observed among the three selected SNPs and neuroblastoma risk in the combined study population.

**Table 1:** Association between selected miRNAs polymorphisms and neuroblastoma risk

Genotype	Cases (*n*=313)	Controls (*n*=762)	*P*^1^	Crude OR (95% CI)	*P*	Adjusted OR (95% CI)^2^	*P*^2^
*miR146a* rs2910164 C>G							
CC	114 (36.42)	272 (35.70)		1.00		1.00	
CG	159 (50.80)	365 (47.90)		1.04 (0.78–1.39)	0.792	1.05 (0.79–1.40)	0.758
GG	40 (12.78)	125 (16.40)		0.76 (0.50–1.16)	0.206	0.76 (0.50–1.16)	0.199
Additive			0.314	0.91 (0.75–1.11)	0.345	0.91 (0.75–1.11)	0.345
Dominant	199 (63.58)	490 (64.30)	0.822	0.97 (0.74–1.27)	0.821	0.97 (0.74–1.28)	0.844
Recessive	270 (87.22)	637 (83.60)	0.134	0.75 (0.51–1.10)	0.135	0.74 (0.51–1.09)	0.125
*miR-149* rs2292832 T>C							
TT	226 (72.20)	542 (71.13)		1.00		1.00	
TC	68 (21.73)	161 (21.13)		1.01 (0.73–1.40)	0.938	1.01 (0.73–1.40)	0.944
CC	19 (6.07)	59 (7.74)		0.77 (0.45–1.33)	0.348	0.78 (0.45–1.34)	0.362
Additive			0.629	0.93 (0.75–1.16)	0.504	0.93 (0.75–1.16)	0.515
Dominant	87 (27.80)	220 (28.87)	0.723	0.95 (0.71–1.27)	0.724	0.95 (0.71–1.27)	0.728
Recessive	294 (93.93)	703 (92.26)	0.337	0.77 (0.45–1.31)	0.338	0.78 (0.45–1.33)	0.353
*miR-196a2* rs11614913 T>C							
TT	89 (28.43)	230 (30.18)		1.00		1.00	
TC	163 (52.08)	377 (49.48)		1.12 (0.82–1.52)	0.477	1.12 (0.82–1.52)	0.478
CC	61 (19.49)	155 (20.34)		1.02 (0.69–1.49)	0.931	1.02 (0.69–1.50)	0.922
Additive			0.738	1.02 (0.84–1.23)	0.848	1.02 (0.84–1.23)	0.840
Dominant	224 (71.57)	532 (69.82)	0.568	1.09 (0.81–1.46)	0.569	1.09 (0.81–1.46)	0.566
Recessive	252 (80.51)	607 (79.66)	0.751	0.95 (0.68–1.32)	0.751	0.95 (0.68–1.32)	0.762

1 χ^2^ test for genotype distributions between neuroblastoma cases and cancer-free controls.

2 Adjusted for age and gender.

### Stratified analysis

Next, participants were stratified in terms of tumor sites, gender, and age. Consistently, as shown in Table 2, there was no significant association for these three pre-miRNAs polymorphisms in subgroups (defined by tumor sites, gender, and age).

**Table 2:** Stratification analysis for the association between selected polymorphism and neuroblastoma susceptibility

Variables	rs2910164 (case/control)		AOR (95% CI)^1^	*P*^1^	rs2292832 (case/control)		AOR (95% CI)^1^	*P*^1^	rs11614913 (case/control)		AOR (95% CI)^1^	*P*^1^
	CC/CG	GG			TT	TC/CC			TT	TC/CC		
Age, months												
≤18	124/283	18/57	0.73 (0.41–1.29)	0.271	101/243	41/97	1.03 (0.67–1.59)	0.890	43/99	99/241	0.96 (0.62–1.47)	0.840
>18	149/354	22/68	0.77 (0.46–1.29)	0.318	125/299	46/123	0.89 (0.60–1.33)	0.575	46/131	125/291	1.22 (0.82–1.82)	0.318
Gender												
Female	127/286	18/54	0.75 (0.42–1.34)	0.328	108/247	37/93	0.93 (0.59–1.45)	0.731	47/101	98/239	0.90 (0.59–1.37)	0.610
Male	146/351	22/71	0.75 (0.45–1.25)	0.271	118/295	50/127	0.99 (0.67–1.46)	0.947	42/129	126/293	1.33 (0.88–1.99)	0.174
Sites of origin												
Adrenal gland	62/637	6/125	0.49 (0.21–1.16)	0.103	50/542	18/220	0.88 (0.50–1.55)	0.668	18/230	50/532	1.19 (0.68–2.09)	0.538
Retroperitoneal	107/637	19/125	0.90 (0.53–1.52)	0.691	91/542	35/220	0.96 (0.63–1.46)	0.833	37/230	89/532	1.04 (0.69–1.57)	0.855
Mediastinum	87/637	12/125	0.70 (0.37–1.32)	0.271	71/542	28/220	0.98 (0.61–1.55)	0.920	28/230	71/532	1.09 (0.69–1.74)	0.706
Others	17/637	3/125	0.88 (0.25–3.05)	0.836	14/542	6/220	1.07 (0.41–2.83)	0.893	6/230	14/532	0.98 (0.37–2.59)	0.966

Abbreviation: AOR, adjusted OR; CI, confidence interval.

1 Adjusted for age and gender, omitting the corresponding stratification factor.

## Discussion

In this three-center case–control study of neuroblastoma, we investigated the association of three well-known pre-miRNAs’ polymorphisms with neuroblastoma risk in Eastern Chinese children. To our knowledge, this is the first replication study for the first time in Eastern Chinese children, regarding such association.

miRNAs could negatively regulate gene expression by binding 3′-UTR of target mRNAs and subsequently affect cell differentiation, proliferation [49]. *miR-146a* rs2910164 G>C, *miR-149* rs2292832 C>T and *miR-196a2* rs11614913 T>C polymorphisms have been reported to be associated with the risk of breast, lung, thyroid, and gastric cancer [50,51]. Further studies found that the *miR-196a2* rs11614913 polymorphism was significantly associated with the developing cancer risk [52,53]. According to recent reports, the *miR-146a, miR-149, miR-196a2* polymorphisms may be genetic predisposing factors in different diseases [33,34,54]. The *miR-146a, miR-149* and *miR-196a2* polymorphisms were significantly associated with the expression of TNF-α, Annexin A1, C-reactive protein, and MTHFR [50]. However, in our previous study in South and North China, no significant association among these three pre-miRNAs polymorphisms (*miR146a* rs2910164, *miR-149* rs2292832, and *miR-196a2* rs11614913) and neuroblastoma susceptibility was observed [25].

Herein, we also observed no significant association of these three pre-miRNAs SNPs with neuroblastoma risk in the Eastern Chinese populations. Several limitations existed in the present study. First, the present study population only included Eastern Chinese individuals, and the results need to be validated in other ethnic groups. Second, the statistical power may be limited due to small sample size. Third, because of the retrospective study, selection and information bias might be unavoidable. Finally, most SNPs only have weak effects on tumor risk [55,56]. Only three polymorphisms were investigated in the present study. Some potentially functional SNPs remain to be assessed in the future.

In conclusion, our study showed that three pre-miRNAs polymorphisms (*pre-miR-146a* rs2910164, *pre-miR-149* rs2292832 and *pre-miR-196a2* rs11614913) are not associated with neuroblastoma risk in Eastern Chinese children. Therefore, additional studies with larger sample size are required to fully understand the role of the three pre-miRNAs’ polymorphisms in neuroblastoma susceptibility.

## Supporting information

**Supplemental Table S1:** Demographic characteristics of neuroblastoma cases and cancer-free controls in eastern Chinese children

**Supplemental Table S2:** Function of selected polymorphisms as predicted by SNPinfo (http://snpinfo.niehs.nih.gov/) software

**Supplemental Table S3:** Association between the three selected polymorphisms and neuroblastoma risk (Divided subjects)

## Abbreviations

CI: confidence interval
GWAS: genome-wide association study
HWE: Hardy–Weinberg equilibrium
miRNA: microRNA
OR: odds ratio
pre-miRNA: precursor miRNA
pri-miRNA: primary miRNA
SNP: single nucleotide polymorphism
UTR: untranslated region
