# Supplementary material for: Association of miR-146a, miR-149 and miR-196a2 polymorphisms with neuroblastoma risk in Eastern Chinese population: a three-center case–control study
Source: Biosci Rep. 2019 Jun 7;39(6):BSR20181907. doi: 10.1042/BSR20181907 (PMC6554217; doi:10.1042/BSR20181907)
Supplement: Supplementary file 1 [file bsr20181907_Supp1.pdf]

Supplemental Table 1. Demographic characteristics of neuroblastoma cases and cancer-free controls in eastern Chinese children

Variables	Cases (n=313)		Controls (n=762)		P^{a}
	No.	%	No.	%	
Age range, month	0.001-132.00		0.001-132.00		0.823
Mean ± SD	29.72 ± 28.21		33.04 ± 30.30		
≤18	142	45.37	340	44.62	
>18	171	54.63	422	55.38	
Gender					0.610
Female	145	46.33	340	44.62	
Male	168	53.67	422	55.38	
Sites of origin					
Adrenal gland	68	21.73			
Retroperitoneal region	126	40.26			
Mediastinum	99	31.63			
Other region	20	6.39			

SD, standard deviation.

[a] Two-sided χ^2 test between neuroblastoma patients and cancer-free controls.

Supplemental Table 2. Function of selected polymorphisms as predicted by SNPinfo (http://snpinfo.niehs.nih.gov/) software

**miRNA**	**rs**	**Chr**	**Allele**	**TFBS**	**Splicing (ESE or ESS)**	**Allele**	**Asian**	**CHB**
miR-146a	rs2910164	5	C/G	Y	Y	G	0.469	0.446
miR-149	rs2292832	2	C/T	Y	Y	C	0.339	0.315
miR-196a2	rs11614913	12	C/T	Y	Y	C	0.427	0.411

TFBS, transcription factor binding sites; ESE, exon splicing enhancer; ESS, exon splicing silencer; CHB, Han Chinese in Beijing, China.

Supplemental Table 3. Association between the three selected polymorphisms and neuroblastoma risk (Divided subjects)

Genotype	Jiangsu province				Anhui province				Wenzhou area			
	Cases (N=158)	Controls (N=426)	AOR (95% CI) [a]	*P* [a]	Cases (N=119)	Controls (N=264)	AOR (95% CI) [a]	*P* [a]	Cases (N=36)	Controls (N=72)	AOR (95% CI) [a]	*P* [a]
miR146a rs2910164 C>G												
CC	46 (29.11)	146 (34.27)	1.00		53 (44.54)	94 (35.61)	1.00		15 (41.67)	32 (44.44)	1.00	
CG	92 (58.23)	208 (48.83)	1.41 (0.93-2.13)	0.103	51 (42.86)	129 (48.86)	0.70 (0.44-1.12)	0.133	16 (44.44)	28 (38.89)	1.23 (0.51-2.96)	0.643
GG	20 (12.66)	72 (16.90)	0.89 (0.49-1.61)	0.691	15 (12.61)	41 (15.53)	0.65 (0.33-1.28)	0.209	5 (13.89)	12 (16.67)	0.88 (0.26-2.96)	0.832
Additive			1.02 (0.78-1.34)	0.867			0.77 (0.56-1.07)	0.116			1.00 (0.57-1.74)	0.988
Dominant	112 (70.89)	280 (65.73)	1.28 (0.86-1.90)	0.230	66 (55.46)	170 (64.39)	0.69 (0.44-1.07)	0.094	21 (58.33)	40 (55.56)	1.12 (0.50-2.54)	0.782
Recessive	138 (87.34)	354 (83.10)	0.71 (0.42-1.22)	0.216	104 (87.39)	223 (84.47)	0.78 (0.41-1.48)	0.449	31 (86.11)	60 (83.33)	0.79 (0.26-2.46)	0.687
miR-149 rs2292832 T>C												
TT	107 (67.72)	309 (72.54)	1.00		90 (75.63)	183 (69.32)	1.00		29 (80.56)	50 (69.44)	1.00	
TC	42 (26.58)	77 (18.08)	1.57 (1.02-2.43)	0.042	21 (17.65)	68 (25.76)	0.63 (0.36-1.09)	0.095	5 (13.89)	16 (22.22)	0.55 (0.18-1.67)	0.292
CC	9 (5.70)	40 (9.39)	0.65 (0.31-1.39)	0.271	8 (6.72)	13 (4.92)	1.24 (0.50-3.12)	0.641	2 (5.56)	6 (8.33)	0.60 (0.11-3.21)	0.547
Additive			1.03 (0.77-1.37)	0.841			0.87 (0.59-1.28)	0.470			0.68 (0.33-1.41)	0.299
Dominant	51 (32.28)	117 (27.46)	1.26 (0.85-1.87)	0.254	29(24.37)	81 (30.68)	0.72 (0.44-1.19)	0.202	7 (19.44)	22 (30.56)	0.56 (0.21-1.49)	0.248
Recessive	149 (94.30)	386 (90.61)	0.59 (0.28-1.24)	0.163	111 (93.28)	251 (95.08)	1.39 (0.56-3.45)	0.481	34 (94.44)	66 (91.67)	0.67 (0.13-3.56)	0.640
miR-196a2 rs11614913 T>C												
TT	50 (31.65)	147 (34.51)	1.00		23 (19.33)	70 (26.52)	1.00		16 (44.44)	13 (18.06)	1.00	
TC	79 (50.00)	199 (46.71)	1.18 (0.78-1.79)	0.436	69 (57.98)	133 (50.38)	1.55 (0.89-2.71)	0.124	15 (41.67)	45 (62.50)	0.26 (0.10-0.69)	0.007
CC	29 (18.35)	80 (18.78)	1.09 (0.64-1.85)	0.762	27 (22.69)	61 (23.11)	1.33 (0.69-2.56)	0.398	5 (13.89)	14 (19.44)	0.29 (0.08-1.03)	0.056
Additive			1.06 (0.82-1.37)	0.660			1.15 (0.83-1.57)	0.404			0.47 (0.24-0.90)	0.022
Dominant	108 (68.35)	279 (65.49)	1.15 (0.78-1.71)	0.477	96 (80.67)	194 (73.48)	1.48 (0.87-2.52)	0.152	20 (55.56)	59 (81.94)	0.27 (0.11-0.67)	0.005
Recessive	129 (81.65)	346 (81.22)	0.99 (0.61-1.58)	0.949	92 (77.31)	203 (76.89)	0.98 (0.58-1.64)	0.926	31 (86.11)	58 (80.56)	0.68 (0.22-2.06)	0.491

AOR, adjusted odds ratio; CI, confidence interval.

[a] Adjusted for age and gender.
